# Socio‐Ecological Significance and Anthropogenic Threats to *Berlinia* (Sol. ex Hook.f., 1849) and *Isoberlinia* (Stapf, 1911) Species in Côte d’Ivoire

**DOI:** 10.1002/ece3.72984

**Published:** 2026-01-21

**Authors:** Sekongo Gbambaly Karim, Soro Bakary, N’ Golo Abdoulaye Koné

**Affiliations:** ^1^ Laboratoire d’Ecologie et de Développement Durable (LEDD), Université Nangui ABROGOUA UFR Sciences de la Nature (UFR SN) Abidjan Côte d’Ivoire; ^2^ Centre de Recherche en Écologie (CRE) Abidjan Côte d’Ivoire; ^3^ Station de Recherche en Écologie du Parc National de la Comoé Abidjan Côte d’Ivoire

**Keywords:** ethnobotany, forest biodiversity conservation, indigenous ecological knowledge, socio‐economic importance, stand structure

## Abstract

*Berlinia* and *Isoberlinia* species are ecologically and socio‐economically important in the Sudanian and Zambezian landscapes of West Africa but are increasingly threatened by overexploitation, habitat loss, and climate stress. In Côte d'Ivoire's Guineo‐Sudanian zone, they provide timber, fuelwood, food, medicinal products, and cultural services. This study asks: How do ecological conditions and human pressures, alongside local ethnoecological knowledge, influence the status, use, and conservation of *Berlinia* and *Isoberlinia* species? This study examined the ethnoecological importance, diversity, spatial distribution, and stand structure of *Berlinia* and *Isoberlinia* species to inform conservation strategies integrating Indigenous knowledge. We surveyed 72 plots (900 m^2^) in protected (Comoé National Park) and unprotected areas, focusing on *Berlinia grandiflora* and *Isoberlinia doka forest stands*, and collected ethnobotanical data from 514 households and 28 key informants across 18 villages. Three species were recorded (*Berlinia grandiflora*, *Isoberlinia doka*, *Isoberlinia tomentosa*). Stands in unprotected areas showed significant declines in density, stem diameter, and basal area, while protected areas remained comparatively intact. The demographic structure of the stands indicates that *I. doka* is characterised by a low proportion of large individuals, resulting from selective illegal overexploitation of the species. In contrast, *B. grandiflora* exhibits a regressive population dynamic, with ageing stands and relatively low levels of regeneration. Ethnobotanical surveys revealed diverse uses (23 medicinal, seven handicraft, two fuelwood, one food) and identified eight main drivers of decline, including deforestation, agriculture, unsustainable logging, bushfires, cashew cultivation, and drought. The results highlight the interplay between ecological status and socio‐economic pressures, emphasising the need for community‐based conservation strategies informed by local knowledge to ensure the long‐term sustainability of these key forest species.

## Graphical abstract

Species of the genera *Isoberlinia* and *Berlinia* are threatened with extinction due to the abusive exploitation of their distribution area including various forms of use. However, their availability and socio‐cultural importance are little known and/or neglected. This socio‐ecological assessment of *Berlinia* and *Isoberlinia* species in Côte d'Ivoire reveals a dynamic interplay between ecological processes and human activities. The data suggest that both *B. grandiflora* and *I. doka* exhibit signs of population regression, evidenced by poor regeneration and their stand structures.

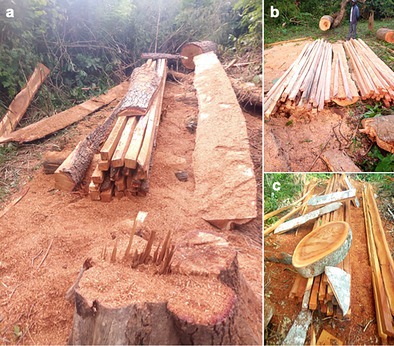

## 1 Introduction

Native to tropical Africa, *Berlinia* and *Isoberlinia* are two genera of dicotyledonous plants belonging to the family Fabaceae, subfamily Caesalpinioideae (Akouègninou et al. 2006; Mackinder and Harris 2006). In West Africa, *Isoberlinia* species occur predominantly between 8° and 13° N latitude (White 1986; Sanon et al. 2019), while *Berlinia* species are primarily distributed in the Sudanian and Guinean zones, with a particular affinity for gallery forests and forest edges (Aubreville 1959). In the Sudanian savannahs of West Africa, the genus *Isoberlinia* comprises two main species: *Isoberlinia doka* Craib & Stapf and *Isoberlinia tomentosa* (Harms) Craib & Stapf (Traoré 2013; Sanon et al. 2019). In Côte d'Ivoire's Guinean and Sudanian savannahs, *Berlinia grandiflora* (Vahl) Hutch. & Dalziel has been recorded (Guillaumet and Adjanohoun 1971).

Over the past decades, interest in species from both genera has increased markedly (Adjahossou et al. 2019). These fast‐growing woody species (Adler 1989) have been identified as promising candidates for domestication in tropical Africa, offering potential to meet the rising demand for timber (Adjahossou et al. 2019). Extensive research has been conducted on *I. doka* dry forests in Benin (Glèlè Kakaï and Sinsin 2009; Goussanou et al. 2017; Adjahossou et al. 2019), Burkina Faso (Bationo et al. 2005; Sanon et al. 2019), and Togo (Dourma et al. 2006, 2012; Dourma 2011), with a focus on stand structure and propagation trials. However, ethnoecological knowledge capable of guiding the sustainable use and diversification of forestry production involving these species remains scarce (Adjahossou et al. 2019). The genus *Berlinia* has received limited research attention in West Africa, apart from a few recent studies in Central Africa (Mapongmetsem 2022; Nganjouong et al. 2022). Its decline is associated with habitat characteristics that hinder seed regeneration, combined with anthropogenic pressures such as logging and recurrent bushfires (Mapongmetsem et al. 2012; Nganjouong et al. 2022).

Ecologically, both genera are significant components of Sudanian and Zambezian landscapes (Dourma et al. 2009; Mapongmetsem et al. 2012). They occur either in pure stands or in association with other large leguminous trees (Glèlè‐Kakaï and Sinsin 2010), and serve as important hosts for edible and medicinal fungi essential to local diets and healthcare systems (Ducousso et al. 2008; Bâ et al. 2011; Vanié‐Léabo 2016). These mycorrhizal associations contribute to soil fertility and ecosystem stability (Smith and Read 2008). Consequently, the conservation of *Berlinia* and *Isoberlinia* is critical not only for biodiversity maintenance but also for sustaining the socio‐economic resilience of Indigenous communities.

From a resource management perspective, species from both genera have gained importance in the context of declining stocks of dense humid and dry forest timber species (Dourma 2011; Louppe 2012; Adjahossou et al. 2019). Once considered of limited economic value, they are now increasingly exploited for their wood, a trend driven by the scarcity of other high‐value species such as iroko ( *Milicia excelsa* (Welw.) C.C.Berg), lingué ( *Afzelia africana* Sm. & Pers.), and caïlcédrat ( *Khaya senegalensis* (Desr.) A. Juss.) (Glèlè‐Kakaï and Sinsin 2010).

Their socio‐cultural value is also substantial, as they provide fuelwood, food, medicinal compounds, and construction materials. For example, *B. grandiflora* is a known source of betulinic acid, which inhibits HIV replication and displays anti‐malarial, anti‐inflammatory, anthelmintic, and antioxidant properties (Yogeeswari and Dharmarajan 2005). Its seeds are consumed in Cameroon and Nigeria (Mapongmetsem 2022). In Benin, the stems of *I. doka* and *I. tomentosa* are widely used as structural timber (Adjahossou et al. 2019). However, such dependence, in the absence of genuine conservation measures, threatens the long‐term sustainability of these resources.

Beyond anthropogenic pressures, climate change poses additional threats. Species from both genera are highly fire‐sensitive, and increasing aridity is likely to exacerbate their vulnerability (Dourma et al. 2012). Consequently, their habitats are shrinking, particularly in Côte d'Ivoire, where studies on these genera are virtually non‐existent. In the Guinean and Sudanian high savannahs of Côte d'Ivoire, they retain significant socio‐economic value, yet overexploitation and habitat degradation are accelerating their decline. Populations are increasingly confined to protected areas, while pure stands in the wild are rare and degraded.

Accurate data on their conservation status in Côte d'Ivoire remain lacking. This is particularly concerning given that logging is officially prohibited north of the 8th parallel, yet selective and semi‐industrial logging persists. Charcoal production in the region is so well‐developed that the output is reputed to be the country's highest in quality (personal observations by NAK).

Understanding the ecological dynamics and drivers of decline is thus crucial for designing effective conservation strategies for *Berlinia* and *Isoberlinia*. We hypothesise that a large‐scale yet largely undocumented exploitation of species from these genera is occurring within their distribution ranges, with no reliable information on its drivers, actors, or extent.

Accordingly, the overarching objective of this study is to understand and mitigate the threats facing taxa of the genera *Berlinia* and *Isoberlinia* in northern Côte d'Ivoire. Specifically, the study aims:

- To assess the diversity and spatial distribution of both genera across their ecological zones.
- To analyse the stand structure of each target species across protected and unprotected areas; and
- To evaluate the impacts of anthropogenic activities on existing stands by examining patterns and intensities of exploitation.

## 2 Material and Methods

### 2.1 Study Area

This study was conducted in the Sudano‐Guinean zone, between meridians 7° and 10°, encompassing the upper central and northern regions of Côte d'Ivoire. The area spans the mesophilic sector of the Guinean domain, as well as the sub‐Sudanese and Sudanese sectors of the Sudanese domain. It is bounded to the north by Burkina Faso and Mali, to the east by Ghana, and to the west by Guinea (Conakry). The study area corresponds to the natural distribution range of *Berlinia* and *Isoberlinia* species (Guillaumet and Adjanohoun 1971).

For logistical efficiency, the study area was virtually subdivided into three principal checkpoints:

1. Northeastern zone**—**encompassing the Bounkani, Gontougo, and Tchologo regions.
2. Northwestern zone**—**comprising the Bagoué, Bafing, Folon, Kabadougou, and Poro regions.
3. Central zone—including the Béré, Hambol, Gbêkê, Iffou, and Worodougou regions.

This subdivision was necessitated by the extensive size of the study area, which required a strategic sampling framework, as well as by variations in topography and the marked cultural heterogeneity of the region. The latter is shaped by the presence of distinct sociolinguistic groups: the Koulango and Lobi peoples predominate in the north‐east; the Baoulé in the central zone; and the Mahouka and Malinké in the north‐west. The far north is predominantly inhabited by the Sénoufo ethnic group.

In the north‐eastern zone, the landscape is characterised by arenaceous plateaux overlying granitic bedrock (Avenard et al. 1971). The central zone consists predominantly of a plateau, whereas the north‐western zone comprises plains interspersed with hills.

The study area comprises a mosaic of savannahs and open dry woodlands. The savannah formations range from wooded and arborescent to shrubby and grassy types. The Guinean savannahs of the mesophilic sector extend northwards to approximately the 8th parallel, which marks both the northern limit of the mesophilic sector and the boundary of the Guinean domain. Within these savannahs, the vegetation includes scattered trees, notably the African fan palm ( *Borassus aethiopum* Mart.), along with numerous gallery forests and relict fragments of dense forest (Guillaumet and Adjanohoun 1971). In the Sudanian zone, the flora of open dry forests is dominated by members of the Fabaceae, Poaceae, Cyperaceae, and Rubiaceae families (Guillaumet and Adjanohoun 1971). In contrast, *B. aethiopum* is absent from the sub‐Sudanese and Sudanese savannahs.

The climate of northern Côte d'Ivoire is characterised by a single rainy season, with peak precipitation occurring in August (Eldin 1971; Kambiré 2010; Soro et al. 2017). The dry season lasts between six and eight months, with its intensity increasing progressively with latitude between parallels 8° and 11° N. Annual rainfall varies between 809.75 and 1160.2 mm (SODEXAM 2024). During the rainy season, relative humidity can reach up to 84%.

### 2.2 Data Collection

Data collection took place from 2022 to 2023 through ecological inventory and ethnobotanical surveys. The ecological component aimed to analyse the structural characteristics of stands of the target species and to map the spatial distribution of each species. The ethnobotanical component focused on documenting and analysing local knowledge, perceptions, and uses associated with these species.

### 2.3 Ecological Inventory

Two sampling methods were employed in this study. The first, an itinerant inventory approach, involved a systematic survey of the entire study area. All forest islands encountered were geo‐referenced using a Garmin GPS 76 device. The second method consisted of fixed‐area surface surveys. Within each of the three predefined checkpoints, as well as in the Comoé National Park (CNP), nine plots measuring 900 m^2^ each were established in two distinct forest types: *Isoberlinia doka* dry forest and gallery forest dominated by *B. grandiflora* .

**FIGURE 1:**
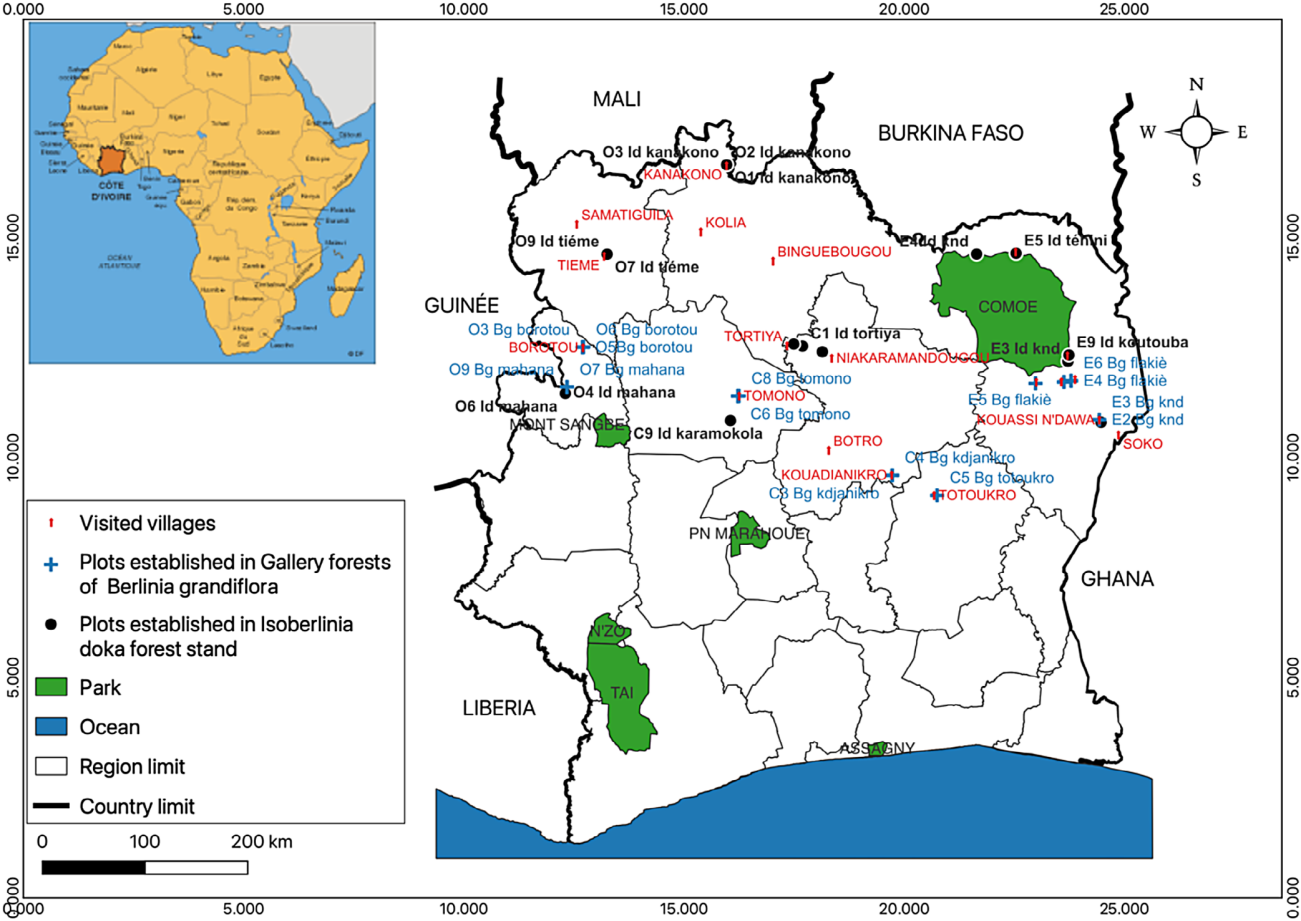
Geographical distribution of surveyed villages for ethnobotanical assessments and sampling plots established across the mesophilic‐Guinean and Sudanian phytogeographical zones of Côte d'Ivoire.

A total of 72 sampling plots (Figure 1) were established across the study area, following a stratified design to capture the ecological variability between the two target vegetation types: gallery forests dominated by *Berlinia grandiflora* and the *Isoberlinia doka* dry forest. The number of plots allocated to each vegetation type was determined proportionally to their surface area within the landscape, ensuring a balanced and representative sampling effort. In the *I. doka* dry forest stand, 36 square plots of 30 × 30 m (900 m^2^) were installed to capture stand structure and disturbance gradients. In contrast, within the gallery forests dominated by *B. grandiflora* , 36 rectangular plots of 45 × 20 m (900 m^2^) were used to better align with the linear physiognomy of these riparian formations. Square plots were deemed unsuitable for gallery forests because of significant edge effects inherent to their shape and the linear forest structure. The sampling rate corresponded to the proportion of the total forested area effectively surveyed through these standardised 900 m^2^ plots. This design ensured sufficient statistical power for structural and ecological analyses.

Within each plot, all species were identified and the number of individuals per species recorded. For each individual, trunk diameter was measured at breast height (approximately 130 cm above ground level). Additionally, the proportion of juvenile individuals, defined as those with a diameter at breast height (dbh) less than 10 cm, was documented.

### 2.4 Ethnobotanical Surveys

Ethnobotanical surveys were conducted in 18 villages distributed across the three designated checkpoints (six villages per checkpoint; see Figure 1). The villages were selected strategically to ensure equidistant coverage of the entire study area. Additionally, village selection was contingent upon the proximity to and local knowledge of at least one of the target species.

Informants were chosen using a combination of simple random sampling and purposive snowball sampling techniques. Simple random sampling entailed selecting a sample of defined size (in this case 30 households per village) by granting the same degree of chance to all elements of the survey population (Houéhanou et al. 2016; Espinosa et al. 2014). This method provides a highly representative population with greater robustness (Litta 2020). In contrast, snowball sampling targeted ‘key informants’‐ individuals identified for their specialised knowledge relevant to the study, such as charcoal producers, sellers, loggers, sawyers, carpenters, and traditional healers. Initial informants selected using the simple random sampling method recommended additional knowledgeable participants within their respective communities, enabling a chain referral process (Bakwaye et al. 2013; Houéhanou et al. 2016). This applies to any adult permanent resident of the village, aged 18 years or older, identified by his community of residence as having a keen knowledge in this study.

Data were so collected from 514 households alongside 28 key informants. Interviews were conducted using a door‐to‐door approach and primarily involved semi‐structured questionnaires that allowed flexibility for emergent, context‐specific questions. The “show‐and‐tell” technique was employed by presenting photographic images or fresh specimens of the plant species to facilitate accurate identification and validation of local knowledge.

Field excursions, guided by local experts, further corroborated species identification and enriched ethnobotanical data. The study encompassed participants of diverse genders, ethnicities, socio‐professional backgrounds, and ages ranging from 18 to over 75 years. The primary focus of data collection was to document Indigenous knowledge regarding the species' uses, cultural importance, and associated practices.

### 2.5 Data Analysis and Statistical Processing

#### 2.5.1 Ecological Data Analysis

##### 2.5.1.1 Spatial Distribution

The GPS data, originally recorded in UTM coordinates during itinerant surveys, were converted into latitude and longitude. Subsequently, a distribution map was produced using QGIS (version 3.26.3).

#### 2.5.2 Population Structure Analysis

The structural parameters employed to characterise the stands of *Berlinia* and *Isoberlinia* comprised density, mean stem diameter, ecological importance value index (IVI), stem distribution in diameter class, Weibull distribution, and stand regeneration capacity.

##### 2.5.2.1 Density

Density (D) refers to the number of standing or living trees per unit area (Tsoumou et al. 2016; Tonga Ketchatang et al. 2017). This metric serves as a robust indicator for assessing forest dynamics and the local potential of plant stands (Koffi 2016). The species density was calculated according to the following formula:

$$D=\frac{\mathrm{ni}}{S}$$

*D* being density of the species in the plot, *ni* the overall number of stems of species ***i*** in the plot and ***S*** the unit area of the plot (S = 0.09 ha).

##### 2.5.2.2 Mean Diameter

The mean diameter is a crucial indicator of the exploitability of woody species. It provides information about the commercial value of the trees.

$$\mathrm{Dm}=\sqrt{\frac{1}{n}\sum_{i=1}^{n}\;{\mathrm{di}}^{2}}$$

With *Dm* the mean diameter, *n* the number of trees found on the plot, and *di* the diameter of the *i*th tree (in cm).

For a tree forked into several main trunks less than 1.3 m in diameter, its diameter (dbh) was determined using the following formula:

$$\mathrm{dbh}=\sqrt{\sum_{i=1}^{n}\;{\text{dbhi}}^{2}}$$

##### 2.5.2.3 Ecological Importance Value Index (IVI) of Cottam and Curtis

The IVI was employed to assess the relative ecological significance of each species within its respective stand. It evaluates the position a species occupies in a plant stand in relation to all other species. Based on the diameter at breast height (dbh) of all major individuals (dbh ≥ 10 cm) of all species recorded in the sampling area, it provides information on the number of individuals, their spatial distribution within the plot, and the proportion of basal area they occupy. The index thereby identifies the most ecologically important species (Beina 2011). It offers a quantified synthesis of a species' overall importance within a stand (Baggnian et al. 2021). The IVI integrates species density, basal area (G), and frequency, and is calculated as the sum of basal area contribution (Cs), relative frequency (Fr), and relative density (d).

$$G=\frac{\pi }{4S}\sum_{i=1}^{n}0.0001{\mathrm{dbh}}^{2}$$

$$\mathrm{Cs}=\frac{\text{Basal area of the species}}{\text{Basal area of}\;\mathrm{all}\;\text{species}\;}\times 100$$

$$\mathrm{Fr}=\frac{\mathrm{Si}}{\mathrm{St}\;}\times 100$$

𝐼𝑉𝐼=Cs+d+Fr

In these equations, *G* is basal area, *S* is the unit area of the plot (*S* = 0.09 ha), dbh is stem diameter at breast height in cm, *Cs* is the basal area contribution of the species, Fr is the observation frequency of the species, *d* is the relative density of the species, and IVI is the ecological importance value index of the species. The IVI varies from 0% to 300%. Species with an IVI ≥ 20% are therefore ecologically important (Litta 2020). An IVI = 300% indicates a monospecific forest stand.

##### 2.5.2.4 Distribution of Stems in Diameter Class

The demographic structure of *Berlinia* and *Isoberlinia* stands was described through the distribution of stems in diameter class. Following the recommendations of Vroh et al. (2015), three classes of stems were established. Mature or adult trees with a diameter greater than 10 cm. Young plants were plants that had their dbh between 5 and 10 cm (5 cm < dbh > 10 cm). The class of recruits was made up of individuals with a diameter of less than 5 cm (5 cm < dbh). Only stems with a dbh greater than or equal to 10 cm were considered for the histograms making to visualise the demographic structure of the stands. To construct these histograms, the observed densities are calculated per diameter class (Glèlè‐kakaï et al. 2016):

$$d_{\text{obsi}}=\frac{n_{i}}{n_{p}S}$$

*d*_obsi_ being the observed density in stems/ha of class *i*; *ni* the number of stems counted for class *i*; *n*_*p*_ the total number of plots considered; *S* the surface area of a plot.

##### 2.5.2.5 Overlay of the Theoretical Weibull Distribution on Diameter Classes

The Weibull distribution is suitable for representing the theoretical structure of stands (Glèlè‐kakaï et al. 2016). It is characterised by great flexibility of use and presents great variability of form depending on the values taken by its theoretical parameters, and thus considers several theoretical distributions, notably normal, exponential, and beta (Bullock and Burkhart 2005; Glèlè‐kakaï et al. 2016).

$$f\left(x\right)=\frac{c}{b}{\left(\frac{x-a}{b}\right)}^{c-1}\exp\left[-{\left(\frac{x-a}{b}\right)}^{c}\right]$$

*f(x)* being the probability density value at point *x*; a is the position parameter; *x* is the diameter; *b* is the scale or size parameter, and *c* is the shape parameter linked to the structure of the diameter classes considered. In our case, *a* is equal to 10 cm because the trees considered have a diameter greater than or equal to 10 cm.

The values of the shape parameter are interpreted as follows:

- *c* < 1: Inverted J or L‐shaped distribution; characteristic of multispecific stands with high regeneration potential.
- *c* = 1: Decreasing exponential distribution; characteristic of stands with high regeneration potential but experiencing survival challenges during transitions between developmental stages.
- 1 < *c*: < 3,6: Positively skewed distribution; characteristic of monospecific artificial stands with a relative predominance of young individuals may also indicate populations with low regeneration potential due to external disturbances, especially in small diameter classes.
- *c* = 3,6: Symmetrical distribution; Characteristic of stands with low regeneration potential caused by external factors.
- *c* > 3,6: Negatively skewed distribution; characteristic of monospecific stands dominated by older individuals, degraded or sometimes nearly extinct populations with very low regeneration potential due to human pressures.

#### 2.5.3 Species' Stand Regeneration Capacity

The species regeneration capacity was assessed using specific indices. The regeneration capacity of a stand provides information about the stability of its forest dynamics. The greater specific regeneration potential of a species, the greater its capacity to colonise large areas. The regeneration potential of a stand (Pr) was calculated as the ratio of juvenile plants (dbh < 10 cm) to the total number of individuals counted in the study area. Similarly, for a given species *i*, its regeneration potential (Pri) is the ratio of juvenile individuals of this species to the total number of individuals of the species. The specific regeneration importance index (SRI) is calculated to determine the regeneration intensity of *Berlinia* and *Isoberlinia* species in relation to the regeneration capacity of the stand. This makes it possible to understand the preponderance of the species studied.

$$\mathrm{Pr}=\frac{\text{Number of juveniles}}{\text{Number of stems in the stand}}\times 100$$

$$\mathrm{Pri}=\frac{\text{Number of juveniles of species}\;i}{\text{Number of stems of species}\;i}\times 100$$

$$\mathrm{IRS}=\frac{\text{Number of juveniles of species}\;i}{\text{Number of juvenile of}\;\mathrm{all}\;\text{species in the stand}\;}\times 100$$

A regeneration potential of a stand or species close to 100% means that the number of juvenile individuals in this forest stand or species is high or the quantity of productive plants is low (Koulibaly et al. 2010). An ISR of between 50% and 100% means that the species has the greatest number of juvenile individuals in the forest stand compared with all the species present there (Ngom et al. 2013).

#### 2.5.4 Statistical Processing

The analysis of variance (ANOVA) was performed using R software (version 4.2.1.) to compare the means of various parameters across the different sub‐sites (protected and unprotected). In the case of non‐normal distribution of variables, non‐parametric Kruskal‐Wallis tests were applied. Significant differences between means were further examined using the Tukey test at a 5% significance level.

#### 2.5.5 Ethnobotanical Data Analysis

##### 2.5.5.1 Level of Knowledge of the Studied Plant Species

The level of knowledge is expressed by the frequency of citation Fc (Malan 2008). This citation frequency corresponds to the number of respondents *n* who mentioned a species out of the total number of informants *N*. This index, based on consensual usage of the species, measures the credibility of the information gathered during the surveys (Schrauf and Sanchez 2008), classifying species as well‐known (50% ≤ Fc ≤ 100%), moderately known (25% ≤ Fc ≤ 50%), or least known (0% ≤ Fc ≤ 25%).

$$\mathrm{fc}=\frac{n}{N}\times 100$$

##### 2.5.5.2 Species' Importance

Key ethnobotanical indices were employed to quantify species' importance, including ethnobotanical use value (UVs), total uses, Cultural Importance Index (CI), and Smith Index (Sa).

##### 2.5.5.3 Intensity of Use of the Species

The intensity of use of a species is determined by its ethnobotanical use value UVs (Tiétiambou et al. 2016). The use value is a basic tool in the selection of species of socio‐economic and cultural interest and subject to strong anthropic pressure (Dossou et al. 2012). This index is obtained using the following formula:

$$\mathrm{UVs}=\sum \frac{\mathrm{Ui}}{N}$$

Where: Ui is the number of uses mentioned by each informant for a species and *N* the total number of informants. A use value greater than 1.2 means that the species is widely used (Kouakou et al. 2020).

##### 2.5.5.4 Total Uses and Use Category

The different uses of the species have been recorded and their numbers added up for each species. The various uses of the species collected are grouped into categories of use. Categories included:

- Food: Consumption of any part of the species, excluding medicinal uses.
- Crafts: Utilisation in housing, furniture, and household items.
- Local Pharmacopoeia: Medicinal, religious, and medico‐magical uses.
- Combustible: Firewood and charcoal production.

##### 2.5.5.5 Cultural Importance Index (CI)

Cultural Importance Index assessed each species' cultural significance. This index is the most objective of the indices based on a consensus of informants because it includes the diversity of uses (Tardío and Pardo‐de‐Santayana 2008). Calculated by summing individual reports of use (UR) per species, divided by the total respondents (N):

$$\mathrm{CI}=\sum \frac{\mathrm{UR}}{N}$$

CI ranges from 0 to the total number of use categories, with values ≥ 0.5 indicating high cultural importance.

##### 2.5.5.6 Smith's Index (Sa)

Smith Index is used to assess informants' knowledge of plants and the cognitive importance of each category of use (Litta 2020). Using the frequency, rank, and length of citation lists, the Smith index is an average of the inverse of the rank of an item between several free lists, weighted by the number of items per list. A high value of this index indicates that the item is important to the respondents.

$$\mathrm{Sa}=\left\{\sum \left[\left(\mathrm{Li}-\mathrm{Rj}+1\right)/\mathrm{Li}\right]\right\}/N$$

where Li is the citation list length and Ri is the citation rank. Sa values range from 0 to 1, indicating maximal importance at 1.

##### 2.5.5.7 Similarity of Uses

Jaccard's Index (S) was used to measure similarity of use between the sociolinguistic groups. It was calculated as follows:

$$\text{Jaccard’s}\;Index\;\left(S\right)=\frac{100c}{\left(a+b-c\right)}$$

where *a* and *b* represent unique uses for each group, and *c* represents shared uses. Interpretation of *S* values:

*S* = 0: No shared use; groups are dissimilar.

0 < *S* < 0.5: Low similarity.

0.5 ≤ *S* < 1: High similarity.

*S* = 1: Perfect similarity; groups are identical in cited uses.

Van Cuyck's (2005) chi‐square test was employed to examine the interdependence between Indigenous communities and the ailments reported. The analysis was conducted in R software (version 4.2.1) using citation data. This statistical approach assesses the association between two categorical variables represented as the rows and columns of a contingency table (Hervé 2016).

#### 2.5.6 Integration of Data

Ethnobotanical and ecological datasets were integrated to provide a comprehensive account of population structure and local knowledge relating to potentially threatened species within the *Berlinia* and *Isoberlinia* genera. The analysis entailed a systematic comparison of local knowledge with ecological indicators to assess potential threats and to guide conservation strategies.

## 3 Results

### 3.1 Diversity and Spatial Distribution of *Berlinia* and *Isoberlinia* Species

**FIGURE 2:**
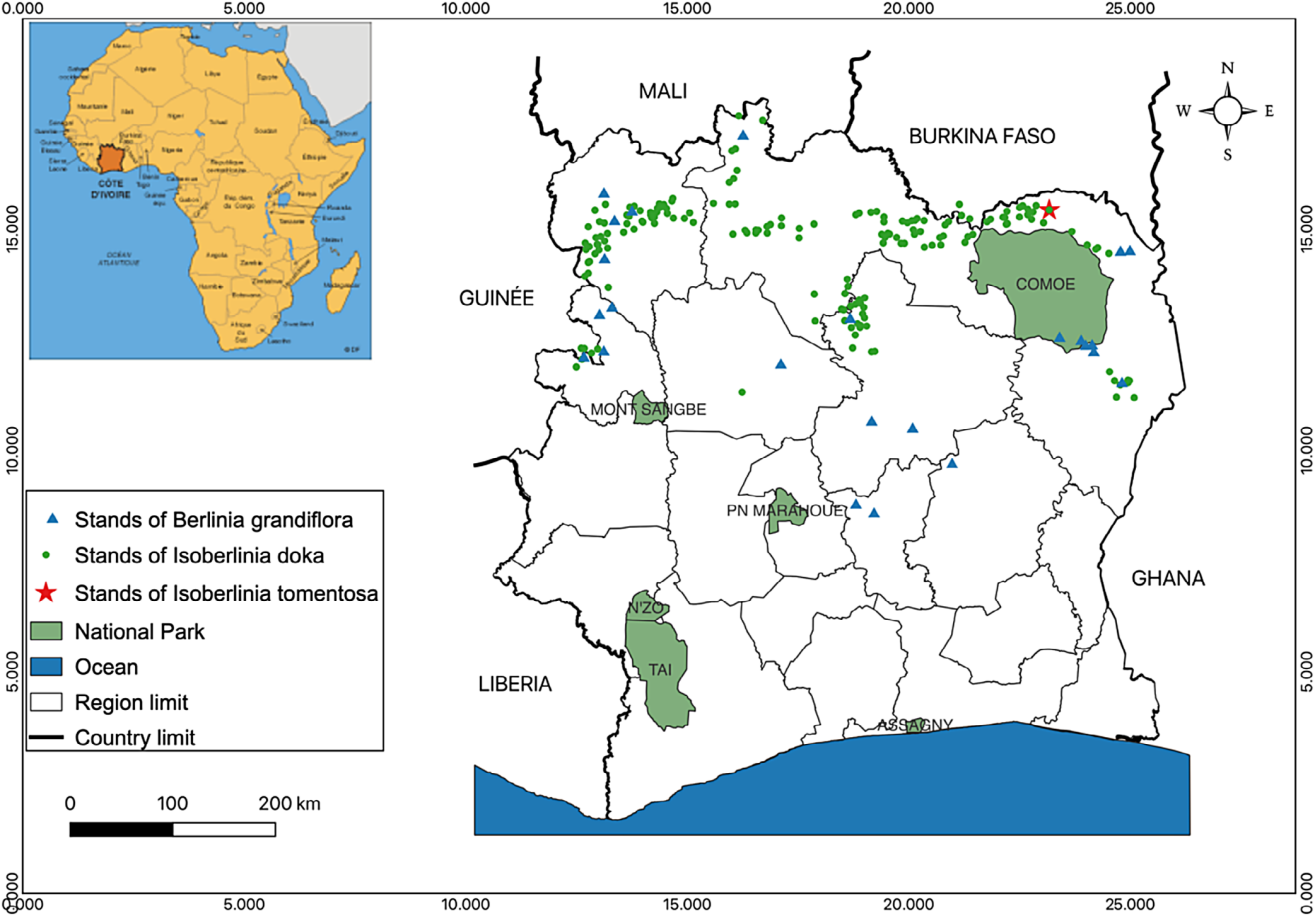
Spatial distribution of *Berlinia grandiflora, Isoberlinia doka*, and *Isoberlinia tomentosa* stands through the mesophilic‐Guinean and Sudanese phytogeographic region of Côte d'Ivoire.

The survey recorded three species belonging to the genera *Berlinia* and *Isoberlinia: Berlinia grandiflora* (Vahl) Hutch. & Dalziel, *Isoberlinia doka* (Craib & Stapf) Baker f., and *Isoberlinia tomentosa* (Harms) Craib & Stapf. The distribution of these species across central and northern Côte d'Ivoire reflects distinct ecological preferences. *Berlinia grandiflora* is widely distributed across the country, with a pronounced concentration in the central region. This species thrives on hydromorphic soils, particularly along rivers and within gallery forests (Figure 2). In contrast, *I. doka* occurs predominantly in the northern regions, from the 7th and 8th meridians to the northern borders with Burkina Faso, Guinea, and Mali. *Isoberlinia tomentosa*, typically co‐occurring with *I. doka* in dry forest islands, is comparatively rare.

### 3.2 Stand Structure Across Species of the Target Genera

#### 3.2.1 Preponderance of Species

In *B. grandiflora* , densities ranged from 123 to 202 stems ha^−1^ (Table 1). *Berlinia grandiflora* displays substantial canopy dominance relative to its basal area contribution (Cs). The contribution to basal area ranged from 52.46% to 75.146% in the natural habitat. In protected areas (CNP), Cs reaches 78.456%. Mean stem diameter of *B. grandiflora* varies between 156 cm and 211 cm. These dendrometric parameters are statistically similar between the different sites, with the exception of density and basal area (*p* < 0.01), which are both higher in the central region. The Importance Value Index (IVI) of *B. grandiflora* consistently exceeded the 20% threshold, confirming its ecological dominance in gallery forests.

**TABLE 1:** Dendrometric characteristics of *Berlinia grandiflora* stands.

Parameters	*Berlinia grandiflora*			
	North‐East	Center	North‐West	CNP
Absolute density (stems/ha)	149.142 ± 44.82 (88.89–211.11)	201.714 ± 44.1 (111.11–233.33)	122.333 ± 33.33 (77.78–188.89)	168.167 ± 58.26 (122.22–311.11)
Relative density (%)	27.166 ± 6.31 (16.39–38.1)	29.315 ± 8.01 (22.22–44.83)	23.642 ± 7.84 (20–44.82)	32.508 ± 5.93 (24.21–43.08)
Basal area (m^2^/ha)	405.864 ± 182.79 (150.03–829.94)	578.223 ± 168.35 (175.61–1073.92)	232.225 ± 101.54 (164.76–349)	493.061 ± 117.6 (396.39–624.58)
Basal area contribution (Cs, %)	61.149 ± 24.79 (15.98–92.54)	75.156 ± 12.9 (55.04–87.33)	52.46 ± 17.96 (34.36–70.28)	78.456 ± 10.59 (59.75–86.89)
Mean diameter (cm)	181.276 ± 61.42 (131.15–307.76)	211.378 ± 55.58 (141.89–296.55)	156.421 ± 42.58 (122.38–182.6)	172.375 ± 10.68 (142.19–263.51)
IVI (%)	175.815 ± 27.07 (137.44–218.13)	187.801 ± 17.52 (157.03–199.78)	110.403 ± 20.58 (88.66–129.59)	198.463 ± 12.73 (177.11–215.64)

*Note:* The values in the table are given in the following order: mean ± standard deviation (minimum – maximum). Sample size was 9 × 900 m^2^ per species and per site.

In *I. doka*, density ranged from 159 to 252 stems ha^−1^ (Table 2), with highest concentrations on the far northern plains and hills (between meridians 9 and 10) and in the CNP. Basal area contribution (Cs) showed no significant difference between protected and unprotected sites. Mean stem diameter and basal area (*p* < 0.01) were both significantly higher in the CNP. The exceptionally high IVI (ranging from 126.056% to 214.629%) indicates strong ecological dominance, suggesting that *I. doka* plays a keystone role in its dry forest structure and function.

**TABLE 2:** Dendrometric characteristics of *Isoberlinia doka* and *Isoberlinia tomentosa* stands.

Parameters	North‐East	Center	North‐West	CNP
***Isoberlinia doka***				
Absolute density (stems/ha)	251.375 ± 122.17 (88.88–466.67)	170.333 ± 30.42 (133.33–211.11)	158.25 ± 67 (100–277.78)	239.506 ± 54.46 (155.55–322.22)
Relative density (%)	48.056 ± 8.77 (38.1–57.69)	34.247 ± 5.66 (26.08–41.37)	52.911 ± 10.95 (40.54–68.57)	39.326 ± 13.79 (10.34–55.03)
Basal area (m^2^/ha)	69.378 ± 34.54 (20.49–107.96)	72.57 ± 8.65 (66.08–82.4)	65.721 ± 18.02 (51.11–88.61)	146.188 ± 63.05 (110.11–325.28)
Basal area contribution (Cs, %)	56.412 ± 16.74 (33.57–79.2)	41.808 ± 15.98 (27.44–59.02)	61.718 ± 21.76 (29.37–75.02)	63.678 ± 13.32 (43.56–78.19)
Mean diameter (cm)	61.81 ± 22.73 (39.28–97.57)	74.648 ± 15.78 (53.4–88.72)	73.515 ± 19.52 (40.75–79.48)	105.569 ± 47.82 (64.25–158.59)
IVI (%)	166.968 ± 21.32 (134.17–199.39)	126.056 ± 16.91 (113.95–145.39)	214.629 ± 30.88 (168.49–233.34)	165.504 ± 22.49 (123.15–186.75)
***Isoberlinia tomentosa***				
Absolute density (in stems/ha)	—	—	—	157.5 ± 53.31 (40–155)
Relative density (%)	—	—	—	40.74 ± 15.9 (8.24–46.27)
Basal area (m^2^/ha)	—	—	—	101.654 ± 91.03 (66.55–272.57)
Basal area contribution (Cs, %)	—	—	—	61.49 ± 16.15 (40.51–78.29)
Mean diameter (cm)	—	—	—	74.97 ± 27.37 (48.91–121.13)
IVI (%)	—	—	—	124.44 ± 31.11 (84.38–158.72)

*Note:* The values in the table are given in the following order: mean ± standard deviation (minimum – maximum). Sample size was 9 × 900 m^2^ per species and per site.

*Isoberlinia tomentosa* occurred at low densities in unprotected habitats (≈22 stems ha^−1^) but was more abundant in the CNP (157 stems ha^−1^). Mean stem diameters were 65.33 cm (unprotected) and 74.97 cm (CNP). Basal area was 18.632 m^2^ ha^−1^ (unprotected) versus 101.654 m^2^ ha^−1^ (CNP) (Table 2). These patterns suggest that *I. tomentosa* benefits disproportionately from protection, possibly due to reduced fire frequency and lower harvesting pressure.

#### 3.2.2 Distribution of Stem Diameters

**FIGURE 3:**
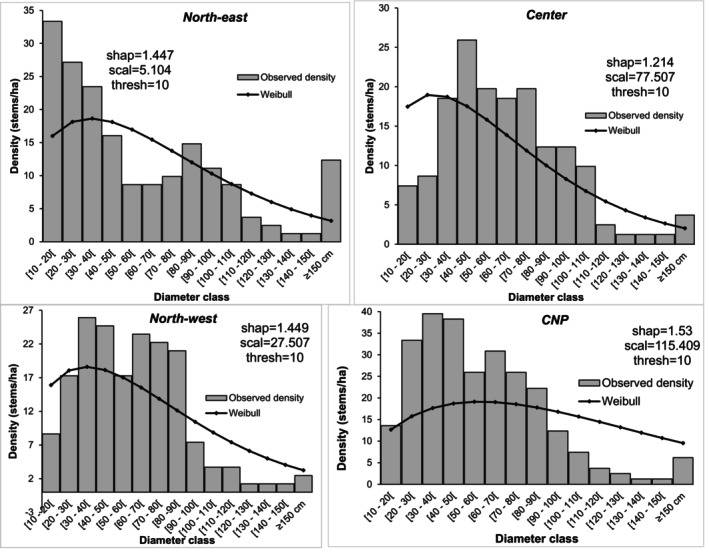
Diameter‐class distributions of stems and their corresponding theoretical Weibull functions for *Isoberlinia doka* across the various regions of the country.

In dry forests of *I. doka*, stems' distribution in diameter class were predominantly “L‐shaped” or “inverted J‐shaped”, reflecting abundant juvenile recruitment (Figure 3). In the north‐east, the pattern was bimodal inverted J‐shaped, whereas the north‐west, central region, and CNP showed slightly bell‐shaped distributions. All these structures fitted Weibull models with shape parameters > 1, typical of monospecific stands with high recruitment rates. Stems of 10–50 cm diameter predominated; > 90% of stems were < 100 cm in diameter, and large stems (> 100 cm circumference) were rare, with < 10 stems ha^−1^.

**FIGURE 4:**
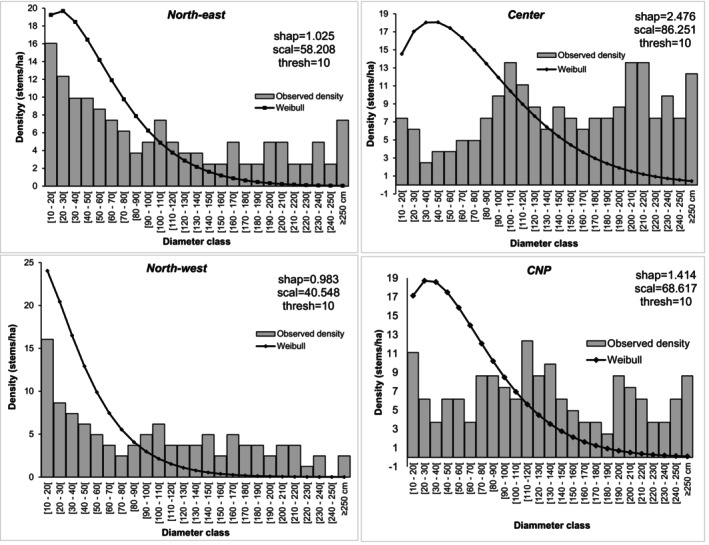
Diameter‐class distributions of stems and their corresponding theoretical Weibull functions for *Berlinia grandiflora* across the various regions of the country.

In stands of *B. grandiflora* , the distribution across diameter classes shows irregular patterns (Figure 4). In the north‐east and north‐west, distributions were “L‐shaped” or “inverted J‐shaped”, dominated by stems < 50 cm in diameter. These distributions fitted Weibull models with shape parameter ≈1, corresponding to a decreasing exponential distribution, indicating populations with high regeneration potential but with a survival problem during the transition between development stages. These distributions are regressive. The vast majority (33.62% in the north‐east and 34.44% in the north‐west) of stems are less than 50 cm in diameter. In the center, the distribution of *B. grandiflora* stems was a “J‐shaped” structure with right‐handed asymmetry. This structure fitted a Weibull distribution with a shape parameter of 2.476. The number of stems increases as the diameter classes increase. The species is characterised by ageing stands with fairly low regeneration. In the CNP, the distribution is asymmetrical and zigzagged. This structure tends towards an ‘i’ distribution. Individuals with a diameter between 110 and 120 cm are the most abundant. The shape parameter of the Weibull distribution was 1.414.

#### 3.2.3 Seminal Regeneration Capacity

In *B. grandiflora* gallery forests, stand‐level seminal regeneration (Pr) did not vary significantly between sites (*p* < 0.01), with values of 16.67% (CNP), 23.08% (north‐east), 28.2% (north‐west), and 31.91% (central). Species‐specific regeneration (Pri) ranged from 12.02% to 18.16%, with the specific importance of regeneration (SIR) highest in the CNP (30.77%) (Table 3).

**TABLE 3:** Natural Seedling regeneration of *Berlinia* and *Isoberlinia* species across study sites.

Index	North‐East	Center	North‐West	CNP
***Berlinia grandiflora***				
Stand regeneration potential (Pr, %)	23.08 ± 6.27 (17.64–33.33)	31.91 ± 4.78 (23.4–33.7)	28.20 ± 5.55 (17.78–41.31)	16.67 ± 3.32 (10.1–19.5)
Species regeneration potential (Pri, %)	18.18 ± 5.67 (9.27–31.26)	14.28 ± 4.91 (9.52–19.7)	14.57 ± 3.8 (7.69–19.67)	12.02 ± 6.42 (5.88–21.43)
Specific regeneration importance (SRI, %)	21.18 ± 6.68 (11.11–25.33)	23.07 ± 7.1 (13.33–29.28)	25.76 ± 5.36 (18.32–29.51)	30.77 ± 14.37 (12.76–4.69)
***Isoberlinia doka***				
Stand regeneration potential (Pr, %)	30.36 ± 3.02 (27.58–32.43)	28.57 ± 2.38 (26.19–35.24)	28.57 ± 5.17 (21.43–36.53)	28.86 ± 6.7 (25.45–37.86)
Species regeneration potential (Pri, %)	33.33 ± 9.58 (27.02–41.08)	33.04 ± 11.11 (28.22–51.17)	22.22 ± 11.15 (15.79–37.5)	34.14 ± 10.64 (27.33–51.4)
Specific regeneration importance (SRI, %)	55.56 ± 15.29 (33.34–69.27)	54.56 ± 19.94 (37.22–74.2)	31.58 ± 19.69 (16.67–60.7)	65.11 ± 23.69 (32.79–84.23)
***Isoberlinia tomentosa***				
Stand regeneration potential (Pr, %)	—	—	—	17.79 ± 4.01 (12.22–21.42)
Species regeneration potential (Pri, %)	—	—	—	12.67 ± 5.1 (7.82–16.66)
Specific regeneration importance (SRI, %)	—	—	—	33.33 ± 10.67 (15.38–48.61)

*Note:* The values in the table are given in the following order: mean ± standard deviation (minimum – maximum). Sample size was 9 × 900 m^2^ per species and per site.

In the *I. doka* dry forests, the seminal regeneration of the stands (Pr) varied little statistically (*p < 0.01*). High values were observed on the north‐eastern hills, while low values were in the CNP. The Pri for *I. doka* was 22.22% in the north‐east and 34.14% in the CNP. The specific regeneration importance index IRS was higher in the CNP. For *I. tomentosa* , regeneration in the CNP was 12.67% with an IRS of 33.33% (Table 3).

### 3.3 Patterns and Intensity of the Exploitation of the Target Species Under Anthropogenic Pressure

#### 3.3.1 Local Knowledge of the Target Species

**FIGURE 5:**
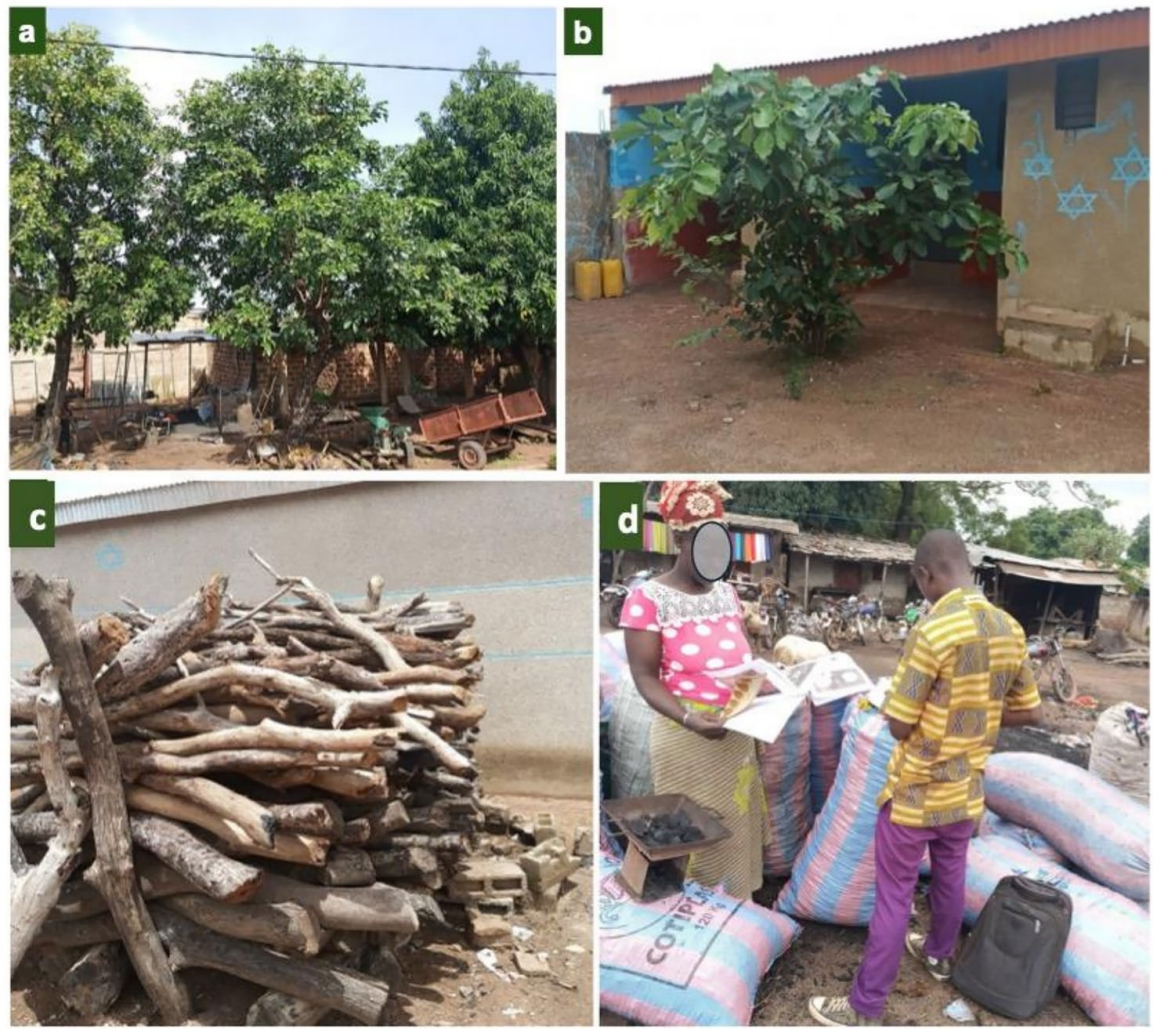
Illustrations of fuel and artisanal uses of *Berlinia grandiflora* and *Isoberlinia doka* through the study region: (a) and (b) *Isoberlinia doka* used as shade trees in surveyed villages; (c) Saving of firewood from *Isoberlinia doka* stems; (d) Marketing of charcoal extracted from the stems of *Berlinia grandiflora*.

Ethnobotanical knowledge of *Berlinia grandiflora*, *Isoberlinia doka*, and *Isoberlinia tomentosa* was widespread across the study sites, with 85.98% of respondents recognising at least one species. Recognition was highest for *B. grandiflora* (52.22%–68.75%) and *I. doka* (28.28%–85.08%) (Table 4), with notable geographical variation. *B. grandiflora* was most widely known in central regions, particularly near the 8th parallel, where it was in high demand for charcoal production and firewood (65.7% citation frequency); whereas *I. doka* dominated in the Sudanese zone, with recognition rates reaching 99% and frequent use as a shade tree (Figure 5). *Isoberlinia tomentosa* was rarely known and often confused with *I. doka*.

**TABLE 4:** Cultural significance (IC), citation frequency (Fc), and ethnobotanical use value (UVs) of *Berlinia grandiflora* and *Isoberlinia doka*.

Zone species	North‐east			Center			North‐west		
	IC	Fc	Uvs	IC	Fc	Uvs	IC	Fc	Uvs
*Berlinia grandiflora*	1.733	52.22	3.34	2.45	68.75	3.134	1.944	62.43	2.769
*Isoberlinia doka*	0.744	48.49	1.522	0.633	28.18	1.92	1.611	85.08	1.688

#### 3.3.2 Species Significance and Use Diversity

Both *B. grandiflora* and *I. doka* had high Use Values (UV > 1.2) and Cultural Importance Index (CI > 0.5) across sites, reflecting their practical and cultural significance (Table 4). Thirty‐two uses were recorded for the two genera: *B. grandiflora* had 13 unique uses, *I. doka* had 4, and both shared 15. These fell into three main categories—traditional medicine, fuel, and handicrafts—with *B. grandiflora* additionally used as food.

These species also serve as an important fodder source for shepherds during the dry season. Additionally, forest islands containing *B. grandiflora* and *I. doka* are preferred sites for collecting edible mushrooms by Indigenous communities. Local populations were well aware that during the rainy season, several mushroom species thrive in the undergrowth of *B. grandiflora* and *I. doka*, with some being highly edible and commonly collected.

#### 3.3.3 Fuel, Food, and Craft Applications

Fuel use was the most frequently cited category, with both species serving as primary sources of firewood and charcoal (Table 5). The sole food‐related use was the production of potash from burnt fruit valves of *B. grandiflora* , employed in traditional soap‐making and in cooking maize dough (locally referred to as *kabato*).

**TABLE 5:** Proportions of fuel, craft and food utilisation of *Berlinia grandiflora* and *Isoberlinia doka*.

Uses	Frequency of citation (%)	
	*Berlinia grandiflora*	*Isoberlinia doka*
Firewood	98.7	91.11
Timber	72.4	29.75
Charcoal	49.67	8.89
Furniture	32.14	5.211
Clean pots	26.95	40.49
Shed	10.71	2.14
Children's shoes	4.22	1.84
Mortars/Pestles	1.94	—
Potash	1.29	—

**FIGURE 6:**
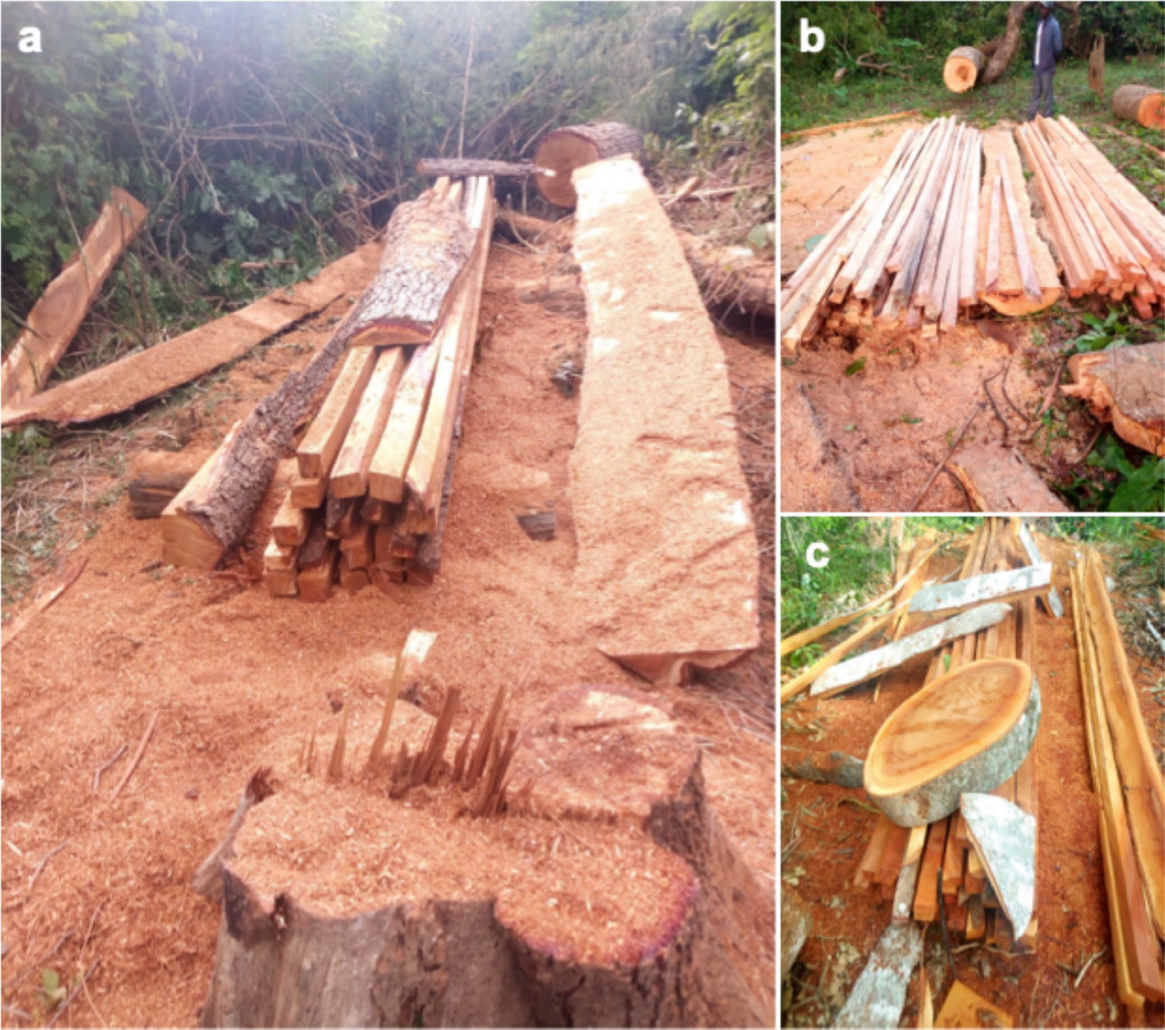
Construction timber sawing of *Isoberlinia doka* (a) and *Berlinia grandiflora* (b, c) woods.

Artisanal uses (seven in total) included timber production (Figure 6), carpentry, pot cleaning, improvised footwear, and shed construction, with two additional uses unique to *B. grandiflora* : crafting mortars and pestles, and using pubescent fruit hairs as a cosmetic pigment.

#### 3.3.4 Medicinal Applications

**FIGURE 7:**
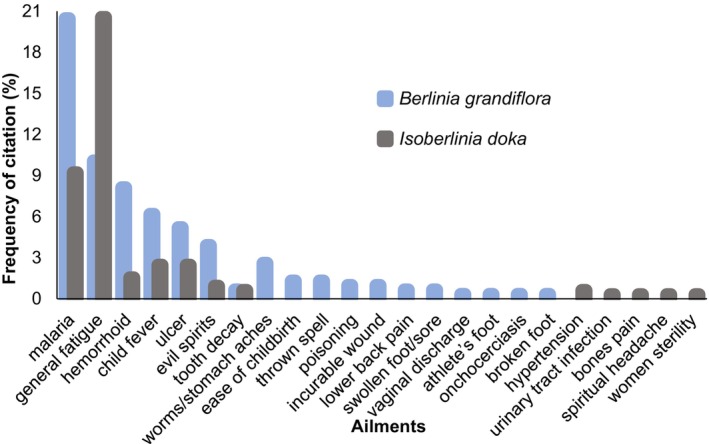
Locally reported diseases treated with *Berlinia grandiflora* and *Isoberlinia doka*, showing vernacular names and citation frequencies across the study areas.

A total of 23 ailments were reported as being treated with the target species, mainly by farmers (87.94%). For *B. grandiflora* , malaria was the most frequently cited treatment (20.45%), followed by haemorrhoids, fatigue, children's fever, and gastric ulcers. For *I. doka*, fatigue ranked first (20.55%), followed by malaria, children's fever, ulcers, and haemorrhoids (Figure 7).

The most commonly used plant parts were bark (53.98%) and leaves (31.41%) for *B. grandiflora* , and leaves (50.09%) for *I. doka*. Decoction was the principal preparation method for both species (72.23% and 90.22% of citations, respectively).

#### 3.3.5 Use Preference and Cognitive Significance

The Smith's Index (Sa) revealed a strong preference for fuel uses in both species (Sa ≥ 0.75). In *B. grandiflora* , timber and malaria treatment ranked as medium‐importance uses (0.5 ≤ Sa < 0.75), whereas furniture making, charcoal production, and children's shoes were of low importance (0.25 ≤ Sa < 0.5). Less common uses such as pot cleaning and potash production had very low importance (Sa < 0.25). For *I. doka*, firewood was the only high‐importance use (Sa = 0.93); fatigue treatment had low importance (Sa = 0.41), and all other uses were marginal (Sa < 0.25).

#### 3.3.6 Sociolinguistic Variation in Medicinal Knowledge

The number of ailments treated with *B. grandiflora* varied from 2 to 11 among sociolinguistic groups, with the Sénoufo reporting the highest diversity. Malaria was the only ailment cited by all seven major groups, though citation frequency varied widely (40% among Baoulé and Lobis, 11% among Malinké). For *I. doka*, ailment diversity ranged from none (Baoulé and Mahouka) to six (Senoufo), with malaria and fatigue most frequently cited. Jaccard index analysis indicated low similarity (< 0.5) in medicinal knowledge between most groups, except for perfect homogeneity (*J* = 1) between Malinké and Koyaga. Chi‐square tests confirmed significant associations between sociolinguistic affiliation and reported ailments for both species. The Chi‐square values were 602.84 and 171.76 respectively for *B. grandiflora* and *I. doka* (*p* < 0.05).

#### 3.3.7 Anthropogenic Pressures

**FIGURE 8:**
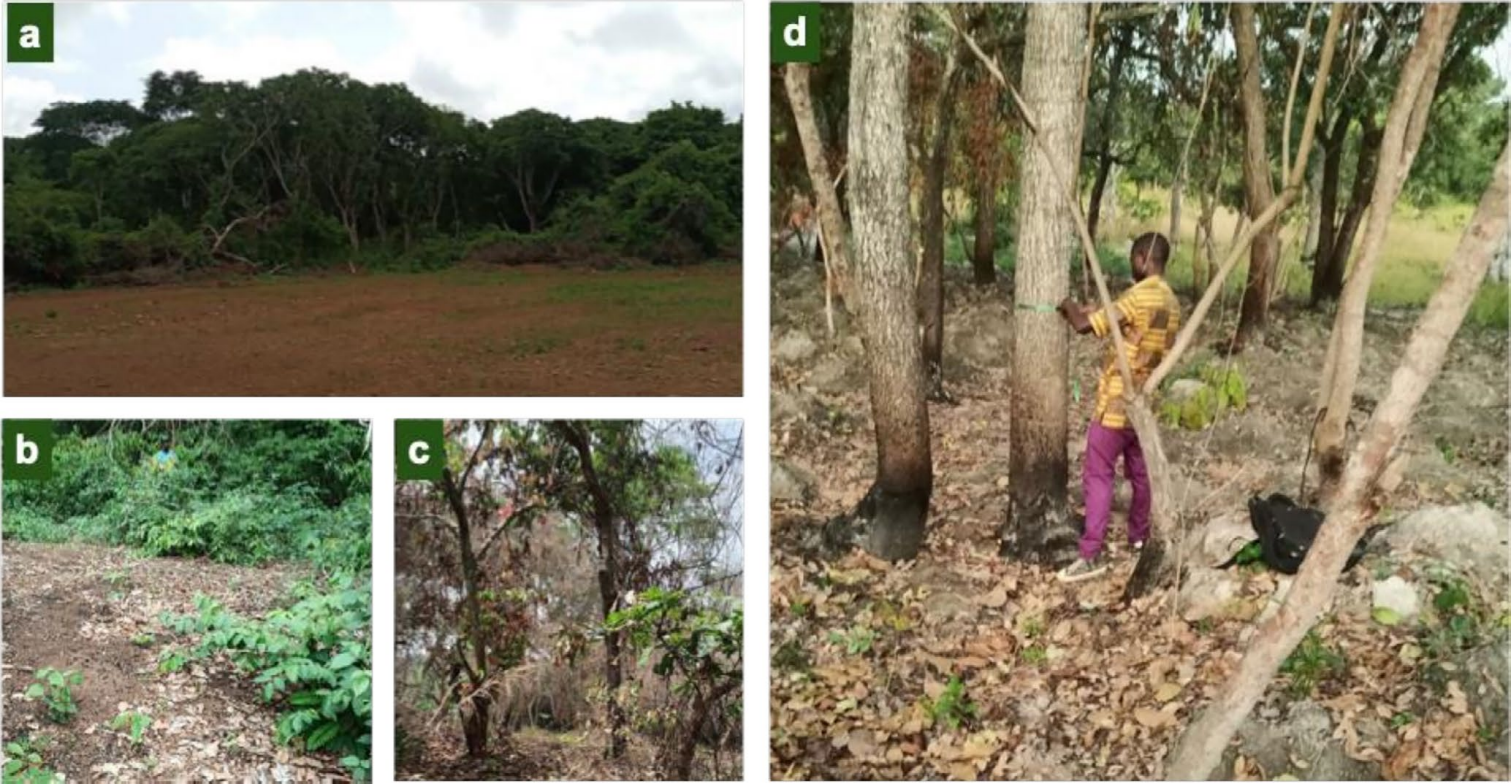
Examples of anthropogenic pressures on *Berlinia* and *Isoberlinia* species. (a) *Berlinia grandiflora* stand cleared for football field construction in Kouadjanikro (Central Côte d'Ivoire); (b) Charcoal production site within a *Berlinia grandiflora* stand in Barakodi (Eastern Côte d'Ivoire); (c) *Berlinia grandiflora* plot recently burned for cashew orchard establishment in Kouadjanikro (Central Côte d'Ivoire); (d) Slash‐and‐burn clearing for yam cultivation within an *Isoberlinia doka* stand near Tortiya (Central‐northern Côte d'Ivoire).

Seven key factors of stands decline were identified by local communities (Figure 8). Deforestation was the most frequently cited (54.44%), followed by agricultural expansion (13.69%), overexploitation for firewood, timber, and charcoal (6.53%), cashew cultivation (4.44%), bushfires from slash‐and‐burn agriculture (2.22%), urbanisation (1.81%), and drought (0.34%).

## 4 Discussion

This study, the first of its kind in Côte d'Ivoire, complements existing data from West Africa on forest galleries and dry forests containing *B. grandiflora* and *Isoberlinia* spp. (Bationo et al. 2005; Sokpon et al. 2006; Fonton et al. 2009; Dourma et al. 2012; Goussanou et al. 2017). A multidisciplinary approach was employed, combining ethnobotanical surveys, ecological inventories, and statistical analyses to assess the stand structure and Indigenous knowledge of *Berlinia* and *Isoberlinia* species within the Sudano–Guinean phytogeographical zone of Côte d'Ivoire. This integrated methodology allowed for a holistic understanding of both the ecological status and sociocultural value of these species, which are increasingly threatened by anthropogenic pressures.

### 4.1 Species Diversity and Population Structures

Three species, *Berlinia grandiflora*, *Isoberlinia doka*, and *I. tomentosa* , were recorded across the savannah and forest islands of the study area. These findings are consistent with the literature (Guillaumet and Adjanohoun 1971; Aké‐Assi 2002), but they also provide a more detailed exploration of savannah ecosystems, where coastal and forest species are absent. Compared with earlier surveys, higher frequencies of *B. grandiflora* and *I. doka* were observed, likely due to (i) a systematic focus on forest islands where these species persist and (ii) the sampling effort.

Diameter structure analyses revealed contrasting levels of disturbance across the studied stands. *Isoberlinia doka* displayed a predominantly “L‐shaped” or inverted J‐shaped diameter distribution, a pattern typically associated with disturbed or selectively logged ecosystems, particularly in unprotected landscapes. Large‐diameter trees were notably rare, mainly owing to illegal logging activities. This scarcity was especially pronounced in areas north of the 8th parallel, where timber harvesting is officially prohibited but nonetheless persists.

The degree of overexploitation is further supported by the values of the Weibull distribution shape parameter. This parameter approached 1, a value generally indicative of near‐monospecific cohorts or uneven recruitment. Such a pattern is likely the consequence of ongoing extraction pressure that is not offset by sufficient natural regeneration of *I. doka*, particularly within communities where the species is traditionally utilised.

In contrast, stands located within protected areas, such as the CNP, exhibited markedly healthier structural profiles. Approximately 8.03% of individuals in these sites exceeded 150 cm in diameter, reflecting reduced disturbance, better age‐class representation, and more effective conservation measures.

*Berlinia grandiflora* exhibited a markedly asymmetrical diameter structure, characterised by a predominance of older, large‐sized individuals. While several stands showed clear signs of disturbance, particularly in the eastern part of the study area, the CNP populations in the central region appeared comparatively more stable. In the central region, 72.06% of individuals exceeded 150 cm in circumference, and 8.82% reached diameters of 400 cm, suggesting the persistence of mature cohorts. However, this structural stability is deceptive. Regeneration remains extremely limited, as juvenile individuals are scarce across sites. This deficit is likely linked to unfavourable habitat conditions and intrinsic seed dispersal constraints typical of gallery‐forest species, which restrict the establishment of seedlings beyond favourable microsites. Despite these limitations, *B. grandiflora* retains a dual regeneration strategy: in addition to sexual reproduction, it can reproduce vegetatively through root suckering, particularly in moist environments. This capacity for vegetative resprouting may partially compensate for poor seedling recruitment, helping sustain local populations in the face of environmental pressures and episodic disturbances.

### 4.2 Ethnobotanical Knowledge and Use

The ethnobotanical survey drew upon a demographically diverse sample (63.28% men and 36.72% women, aged 18–75+), the majority of whom (80.44%) were farmers. This profile reflects the socio‐economic structure of rural communities and the gendered dynamics of knowledge transmission in the region. The underrepresentation of women, likely influenced by survey timing, cultural norms, and gendered household responsibilities, suggests that important knowledge may remain under‐documented. Their perspectives are essential and should be the focus of more targeted future investigations.

Local identification and use of the studied species were common across the landscape. Approximately 86% of respondents were able to recognise at least one species using vernacular names, although several distinct species were frequently grouped under a single ethno‐species. The origins of these names varied among ethnic groups; for instance, the term « *Pka Pka »*, used by the Baoulé and Koyaga ethnic groups, mimics the characteristic sound of fruit dehiscence, illustrating the role of sensory cues in vernacular naming systems.

Ethnobotanical knowledge displayed clear geographic variation. *Berlinia grandiflora* was widely recognised across all regions surveyed, whereas *Isoberlinia doka* was more familiar to respondents in the northern zone. By contrast, *I. tomentosa* was often confused with *I. doka*, resulting in fewer independent citations. This pattern highlights the challenges posed by morphological similarity and emphasises the value of field‐based training to improve species‐level recognition in ethnobotanical research. Overall, the spatial distribution of knowledge mirrored the ecological preferences of each taxon: *B. grandiflora* occurred primarily in riparian forest corridors, while *Isoberlinia* species were associated with the drier savannah landscapes.

### 4.3 Usage Categories and Socioeconomic Value

Both genera are integral to local livelihoods, providing fuelwood, timber, charcoal, medicine, and food. *B. grandiflora* had 28 reported uses across four categories—fuel, craftsmanship, food, and medicine—with medicinal applications (notably for malaria and fatigue) representing the largest share. *I. doka* and *I. tomentosa* had 19 and fewer uses, respectively, primarily for fuel, construction, and traditional medicine; however, shared ethno‐taxonomic status complicates separate use assessments.

Medicinal applications, although numerous, were not deemed highly significant by quantification indices, possibly due to secrecy surrounding traditional knowledge. Common ailments treated include malaria, haemorrhoids, and dental issues, which reflect high local prevalence and hospitalisation rates.

Timber and fuel use dominate due to species' growth characteristics. Both species exhibit rapid annual diameter growth (up to 23.9 mm for *I. doka*), far exceeding averages for other tropical trees (Adler 1989). This rapid growth enhances their desirability among sawyers, contributing to overharvesting.

Artisanal preferences vary regionally. For example, *B. grandiflora* is preferred in Kouassi N'dawa for charcoal due to its quality, whereas *I. doka* is avoided for this use because of its oily, toxic smoke. All three species are valued as dry‐season fodder for livestock and wild fauna, including elephants, providing critical green resources during resource‐scarce periods.

Symbiotic associations with edible and medicinal fungi are another important ecosystem service. Ducousso et al. (2008) and Oumarou et al. (2019) documented 70 mushroom species associated with *I. doka* and 35 with *B. grandiflora* , highlighting their role in forest regeneration and local food security.

### 4.4 Threats to Biodiversity and Conservation Gaps

Seven primary anthropogenic threats were identified: deforestation, slash‐and‐burn agriculture, cashew and mango expansion, firewood and charcoal production, bushfires, urbanisation, and gold panning. Cashew cultivation, encouraged by government policy of price increases during the periods 1994–2000 and 2014–2017 (Ruf et al. 2019), caused substantial land conversion to orchards. Mango farming in regions such as Ferkessédougou and Korhogo followed similar dynamics.

Uncontrolled bushfires significantly impede regeneration. Young *I. doka* and *B. grandiflora* seedlings are highly fire‐sensitive, and repeated fires reduce regeneration and timber quality by causing trunk deformations and rot. Fire‐impacted stands lose productivity and ecological functionality.

Despite their socio‐economic and ecological importance, *Berlinia* and *Isoberlinia* species lack formal or informal protection. Unlike culturally protected species such as *Vitellaria paradoxa* (*si yiri*) or *Parkia biglobosa* (Néré), they are not subject to taboos or traditional restrictions, leaving them highly vulnerable.

### 4.5 Conservation Implications

The findings of this study underscore an urgent need for targeted conservation strategies aimed at safeguarding *Berlinia grandiflora*, *Isoberlinia doka* and *Isoberlinia tomentosa* within the Sudano‐Guinean zone of Côte d'Ivoire. The combined evidence of overexploitation, poor regeneration, habitat fragmentation, and extensive fire and agricultural pressures indicates that current population dynamics are unsustainable. Without immediate intervention, the ecological integrity and socio‐economic value of these species, already diminished in many parts of West Africa, risk further and potentially irreversible decline.

First, the contrasting population structures observed between protected and unprotected areas highlight the effectiveness of formal conservation measures. Stands within the CNP exhibited healthier diameter‐class distributions and lower disturbance levels, suggesting that extending or strengthening protection outside the park could significantly enhance population resilience. Establishing community‐managed conservation zones, integrating these species into local land‐use plans, and enforcing existing logging restrictions particularly north of the 8th parallel should be prioritised.

Second, the notable regeneration deficit of *B. grandiflora* and the selective logging of *I. doka* call for active restoration strategies. Enrichment planting, nursery propagation using both seedlings and vegetative suckers, and the protection of natural regeneration sites from fire and grazing pressures could accelerate population recovery. Promoting assisted natural regeneration (ANR) within forest islands and riparian corridors may be particularly beneficial, given the species' ecological niches and dispersal limitations.

Third, the strong socio‐economic dependence on these taxa, especially for timber, fuelwood, charcoal, and medicine, necessitates sustainable‐use frameworks. Developing village‐level management plans, coupled with training on non‐destructive harvesting techniques and alternatives to fuelwood extraction, would help reduce pressure on wild populations. The high growth rates of *Isoberlinia* spp. also present an opportunity for establishing small‐scale agroforestry or woodlot systems to supply local energy needs while decreasing reliance on natural stands.

Fourth, widespread bushfires represent a critical threat that can be mitigated through strengthened local fire governance. Participatory fire management integrating early dry‐season burns, firebreak maintenance, and community fire surveillance should be incorporated into conservation planning. Considering the cultural and economic drivers of fire use in savannah landscapes, engagement with farmers and herders is essential to ensure the adoption of sustainable burning practices.

Finally, the absence of traditional or institutional protection for these species contrasts sharply with the cultural safeguarding of other key savannah trees, such as *Vitellaria paradoxa* and *Parkia biglobosa* . Promoting awareness of the ecological, economic, and cultural roles of *Berlinia* and *Isoberlinia* species could catalyse community‐led conservation norms. Reinforcing local knowledge systems, especially among women whose underrepresentation in surveys suggests untapped perspectives, may further strengthen the social foundations for species protection.

Overall, this study provides baseline data that can inform national conservation policies, sustainable forest management initiatives, and community‐based strategies in northern Côte d'Ivoire. Ensuring the long‐term viability of *Berlinia* and *Isoberlinia* species will require integrated approaches that jointly address ecological constraints, socio‐economic dependencies, and the pervasive anthropogenic pressures that shape these savannah landscapes.

## 5 Conclusion

This socio‐ecological assessment of *Berlinia* and *Isoberlinia* species highlights the intricate interplay between ecological processes and human activities. These species provide essential ecological functions and support local livelihoods, yet they are increasingly threatened by deforestation, unsustainable land use, and inadequate conservation frameworks. Our data indicate that both *B. grandiflora* and *I. doka* show signs of population decline, reflected in poor regeneration and overexploitation. These trends are most pronounced in unprotected landscapes, whereas protected areas, such as CNP, serve as effective models for sustainable conservation.

Based on observed density and regeneration patterns, conservation efforts should prioritise the protection of habitats where these species are most concentrated—particularly the Central region for *B. grandiflora* and the far North for *I. doka* and *I. tomentosa* . Such efforts must integrate ecological management with community‐based conservation approaches. Initiatives including environmental education, reforestation, promotion of agroforestry, and the cultural valorisation of Indigenous species can facilitate sustainable use. Moreover, incorporating traditional knowledge into national conservation policies could enhance both biodiversity protection and the resilience of rural communities.

By synthesising ecological and ethnobotanical insights, this study emphasises the urgent need for targeted conservation interventions that account for the socio‐economic realities of the rural populations dependent on these critical species.

## Author Contributions

**Sekongo Gbambaly Karim:** data curation (equal), formal analysis (equal), investigation (equal), methodology (equal), software (equal), writing – original draft (equal), writing – review and editing (equal). **Soro Bakary:** conceptualization (equal), methodology (equal), supervision (equal), validation (equal), visualization (equal). **N' Golo Abdoulaye Koné:** conceptualization (equal), data curation (equal), formal analysis (equal), funding acquisition (equal), project administration (equal), resources (equal), supervision (equal), validation (equal), visualization (equal), writing – review and editing (equal).

## Funding

This work was funded by the German Academic Exchange Service DAAD (through the Climate and Environment Center “Future African Savannas_AFAS”) from funds of the German Federal Foreign Office.

## Ethics Statement

The informed consent was obtained verbally by each survey participant prior to the interview; participants were informed of their right to participate voluntarily or to refuse.

## Consent

All participants in this study gave oral consent, and all data are anonymised.

## Conflicts of Interest

The authors declare no conflicts of interest.

## Data Availability

All the datasets used and/or analysed during the current study are submitted as Supporting files.

## References

[ece372984-bib-0001] Adjahossou, S. G. C. , D. T. Houéhanou , M. Toyi , et al. 2019. “Dépendance Socioculturelle des Connaissances Locales des Usages de *Isoberlinia* spp. au Moyen‐Bénin, Afrique de l'Ouest.” Bois et Forêts Des Tropiques 339: 33–43. 10.19182/bft2019.339.a31702.

[ece372984-bib-0002] Adler, D. 1989. “Natural Forest Increment, Growth and Yield.” In Wong J. L. G. and Dunn R. M. Ghana Forest Inventory Project Seminar Proceedings 29–3 March 1989 Overseas Development Administration (UK)/Ghana Forestry Department. pp 47–52.

[ece372984-bib-0003] Aké‐Assi, L. 2002. Flore de la Côte D'ivoire: Catalogue Systématique, Biogéographique et Ecologie. Vol. 58, 1–401. Conservatoire et Jardin Botanique de Genève.

[ece372984-bib-0004] Akouègninou, A. , W. J. der Van Burg , L. J. G. Van Maesen , et al. 2006. Flore Analytique du Bénin, 1034. Backhuys Publishers.

[ece372984-bib-0005] Aubreville, A. 1959. Flore Forestière de Côte D'ivoire. Vol. 1. 2ème Édition ed, 269. Centre Technique Forestier Tropical.

[ece372984-bib-0006] Avenard, J. M. , M. Eldln , G. Girard , et al. 1971. Le Milieu Naturel de la Côte D'ivoire, 9–72. Mémoires ORSTOM.

[ece372984-bib-0007] Bâ, A. , R. Duponnois , M. Diabaté , and B. Dreyfus . 2011. Les Champignons Ectomycorhiziens des Arbres Forestiers en Afrique de l'Ouest: Méthodes D'étude, Diversité, Écologie, Utilisation en Foresterie et Comestibilité. IRD Éditions. 10.4000/books.irdeditions.10404.

[ece372984-bib-0008] Baggnian, I. , Y. M. Mahamadou , and T. Adam . 2021. “Analyse de la Vulnérabilité des Ressources Végétales Ligneuses: Cas du Département de Guidan‐Roumdji, Niger.” Journal of Agriculture and Veterinary Science 14, no. 9: 29–42.

[ece372984-bib-0009] Bakwaye, F. N. , C. Termate , K. Kibungu , and P. Van Damme . 2013. “Identification et Importance Locale des Plantes Médicinales Utilisées Dans la Région de Mbanza‐Ngungu, République Démocratique du Congo.” Bois et Forêts Des Tropiques 316, no. 2: 63–77.

[ece372984-bib-0010] Bationo, B. A. , S. J. Ouédraogo , F. Pallo , and I. J. Boussim . 2005. “Régénération Naturelle *D'Isoberlinia Doka* Craib & Stapf Dans la Forêt Classée de Nazinon (Burkina Faso).” Cahiers D'études et de Recherches Francophones/Agricultures 14, no. 3: 297–304.

[ece372984-bib-0011] Beina, B. 2011. “Diversité Floristique de la Forêt Dense Semi‐Décidue de Mbaïki, République Centrafricaine: Étude Expérimentale de L'impact de Deux Types D'intervention Sylvicole.” Thèse de Doctorat École Doctorale Science et Santé Unité Dynamiques des Systèmes Anthropisés (JE 2532). Université de Picardie Iules Verng France, 226 p.

[ece372984-bib-0012] Bullock, B. P. , and H. E. Burkhart . 2005. “Juvenile Diameter Distributions of Loblollypine Characterized by the Two‐Parameter Weibull Function.” New Forests 29: 233–244. 10.1007/s11056-005-5651-5.

[ece372984-bib-0013] Dossou, M. E. , G. L. Houessou , O. T. Lougbegnon , A. H. B. Tente , and J. T. C. Codjia . 2012. “Étude Ethnobotanique des Ressources Forestières Ligneuses de la Forêt Marécageuse D'Agonvè et Terroirs Connexes au Bénin.” Tropicultura 30, no. 1: 41–48.

[ece372984-bib-0014] Dourma, M. 2011. “Les Forêts Claires à *Isoberlinia doka* Craib & Stapf et *I. tomentosa* (Harms) Craib & Stapf (Fabaceae) en Zone Soudanienne du Togo: Écologie, Régénération Naturelle et Activités Humaines.” Acta Botanica Gallica 158, no. 1: 141–144.

[ece372984-bib-0015] Dourma, M. , A. K. Guelly , K. Kokou , et al. 2006. “Multiplication par Drageonnage *D'Isoberlinia Doka* et *I. tomentosa* au Sein des Formations Arborées du Nord‐Togo.” Bois et Forêt Des Tropiques 289, no. 3: 49–57.

[ece372984-bib-0016] Dourma, M. , K. Wala , R. Bellefontaine , K. Batawila , A. K. Guelly , and K. Akpagana . 2009. “Comparaison de L'utilisation Des Ressources Forestières et de la Régénération Entre Deux Types de Forêts Claires à *Isoberlinia* au Togo.” Bois et Forêt Des Tropiques 302, no. 4: 5–19. 10.19182/bft2009.302.a20400.

[ece372984-bib-0017] Dourma, M. , K. Wala , K. A. Guelly , et al. 2012. “Typologie, Caractéristiques Structurales et Dynamique des Faciès Forestiers Fragiles à *Isoberlinia* spp. en vue de Leur Gestion au Togo.” Bois et Forêts Des Tropiques 313, no. 3: 19–33. 10.19182/bft2012.313.a20494.

[ece372984-bib-0018] Ducousso, M. , H. Ramanankierana , R. Duponnois , et al. 2008. “Mycorrhizal Status of Native Trees and Shrubs From Eastern Madagascar Littoral Forests With Special Emphasis on One New Ectomycorrhizal Endemic Family, the Asteropeiaceae.” New Phytologist 178: 233–238. 18371004 10.1111/j.1469-8137.2008.02389.x

[ece372984-bib-0019] Eldin, M. 1971. “Le Climat.” In Le Milieu Naturel de la Côte D'ivoire, edited by J. M. Avenard , M. Eldin , G. Girard , and J. Sircoulon , vol. 50, 161–262. Mémoires ORSTOM.

[ece372984-bib-0020] Espinosa, M. M. , I. G. C. Bieski , and D. T. O. Martins . 2014. “Sampling in Ethnobotanical Studies of Medicinal Plants: 197–212.” In Methods and Techniques in Ethnobiology and Ethnoecology, Springer Protocols Handbooks. Humana Press.

[ece372984-bib-0021] Fonton, N. H. , C. C. Yabi , J. Z. Dah‐Dovonon , F. K. Adoko , and T. Dotchamou . 2009. “Modélisation du Volume du fût D'arbre Pour une Gestion Durable des Écosystèmes Forestiers Soudaniens.” Bois et Forêts Des Tropiques 300, no. 2: 95–100.

[ece372984-bib-0022] Glèlè Kakaï, R. , and B. Sinsin . 2009. “Structural Description of Two *Isoberlinia* Dominated Communities in the Wari‐Maro Forest Reserve (Benin).” South African Journal of Botany 75, no. 1: 43–51. 10.1016/j.sajb.2008.07.003.

[ece372984-bib-0023] Glèlè‐kakaï, R. , W. Bonou , and A. M. Lykke . 2016. “Approche Méthodologique de Construction et D'interprétation.” Annales Des Sciences Agronomiques 20: 99–112.

[ece372984-bib-0024] Glèlè‐Kakaï, R. , and B. Sinsin . 2010. “Description de *Isoberlinia* spp. Caesalpiniaceae.” In Biodiversity Atlas of West Africa Vol 1, edited by B. Sinsin and D. Kampmann , BIOTA.

[ece372984-bib-0025] Goussanou, C. A. , B. A. Tente , G. Akouehou , V. K. Salako , R. L. Glele‐Kakaï , and B. A. Sinsin . 2017. “Structural and Spatial Patterns of *Isoberlinia* Species in a Disturbed Community Forest (Benin, West Africa).” Kastamonu Üniversitesi Orman Fakültesi Dergisi 17, no. 2: 225–237.

[ece372984-bib-0026] Guillaumet, L. , and E. Adjanohoun . 1971. “La Végétation de la Côte d'Ivoire.” In: Le Milieu Naturel de la Côte d'Ivoire. Mémoire ORSTOM.

[ece372984-bib-0027] Hervé, M. 2016. “Aide‐Mémoire de Statistique Appliquée à la Biologie.” Construire son Étude et Analyser les Résultats à L'aide du Logiciel R. Version 2016. https://cran.r.project.org.

[ece372984-bib-0028] Houéhanou, D. T. , A. E. Assogbadjo , F. J. Chadare , S. Zanvo , and B. Sinsin . 2016. “Approches Méthodologiques Synthétisées des Études D'ethnobotanique Quantitative en Milieu Tropical.” Annales Des Sciences Agronomiques 20: 187–205.

[ece372984-bib-0029] Kambiré, B. 2010. “L'agriculture Vivrière du Nord‐Est Ivoirien en “Régression”: un Danger Pour les Centres Urbains Ivoiriens.” Revue de Géographie Tropicale et D'environnement 2: 40–53.

[ece372984-bib-0030] Koffi, K. A. D. 2016. “Dynamique de la Végétation et Valeurs de Conservation des Espaces Anciennement Cultivés du Parc National D'azagny (Sud de la Côte d'Ivoire).” Thèse de Doctorat, Université Félix‐Houphouët Boigny (Abidjan, Côte d'Ivoire), 233 p.

[ece372984-bib-0031] Kouakou, Y. B. , M. D. Kougbo , A. S. Konan , D. F. Malan , and A. Bakayoko . 2020. “Usages Traditionnels et Disponibilité des Plantes Exploitées Dans L'artisanat Chez les Populations Koulango et Lobi De La Périphérie Est Du Parc National De La Comoé, Côte D'ivoire.” European Scientific Journal 16, no. 9: 31–57. 10.19044/esj.2020.v16n9p295.

[ece372984-bib-0032] Koulibaly, A. V. , N. F. Kouamé , D. Traoré , and S. Porembski . 2010. “Structure et la Régénération de la Végétation Ligneuse, le Long de Transects Forets Savanes, Dans la Région de la Réserve de Lamto (Côte D'ivoire).” Annale Botanique D'afrique de L'ouest 6: 56–72.

[ece372984-bib-0033] Litta, A. L. 2020. “Disponibilité des Plantes Utilisées Traditionnellement Chez les Agni du Centre‐Est et Nord‐Est de la Côte d'Ivoire.” Thèse de Doctorat, Université Nangui Abrogoua, Abidjan Côte d'Ivoire. 255 p.

[ece372984-bib-0034] Louppe, D. 2012. “ *Isoberlinia doka* Craib & Stapf.” In Ressources Végétales de L'Afrique Tropicale, edited by R. H. M. J. Lemmens , D. Louppe , and A. A. Oteng‐Amoako , 465–470. Fondation PROTA.

[ece372984-bib-0035] Mackinder, B. A. , and D. J. Harris . 2006. “A Synopsis of the Genus Berlinia (Leguminosae‐Caesalpinioideae).” Edinburgh Journal of Botany 63, no. 2: 161–182.

[ece372984-bib-0036] Malan, D. F. 2008. “Utilisations Traditionnelles des Plantes et Perspective de Cogestion des Aires Protégées de Côte d'Ivoire: Cas du Parc National des îles Ehotile (Littoral est de la Côte d'Ivoire).” Thèse de Doctorat. Université Abobo‐Adjamé Abidjan, Côte d'Ivoire, 31: 194 p.

[ece372984-bib-0037] Mapongmetsem, P. M. 2022. “Ethnobotany and Demography of *Berlinia gran*diflora in the Guinean Savannah Highlands of Cameroon.” Research Journal of Agriculture and Forestry Sciences.

[ece372984-bib-0038] Mapongmetsem, P. M. , V. N. Kapchie , and B. H. Tefempa . 2012. “Diversity of Local Fruit Trees and Their Contribution in Sustaining the Rural Livelihood in the Northen Cameroon.” Ethiopian Journal of Environmental Studies and Management 5, no. 1: 32–46.

[ece372984-bib-0039] Nganjouong, J. K. , R. Tsobou , G. Fawa , Z. Oumarou , and B. Loura . 2022. “Multiplication Végétative de *Berlinia* Grandiflora par Marcottage Aérien Dans les Hautes Savanes Guinéennes de l'Adamaoua, Cameroun.” Afrique Science 21, no. 3: 15–27.

[ece372984-bib-0040] Ngom, D. , T. Fall , O. Sarr , S. Diatta , and L. E. Akpo . 2013. “Caractéristiques Écologiques du Peuplement Ligneux de la Réserve de Biosphère du Ferlo (Nord Sénégal).” Journal of Applied Biosciences 65, no. 1: 644.

[ece372984-bib-0041] Oumarou, H. , D. P. Perez , B. Moussa , I. Dahiratou , A. M. Rosas , and I. Félix . 2019. “Écologie des Champignons Supérieurs du Parc National du W du Niger (Afrique de l'Ouest). Quels Facteurs Expliquent Leurs Distributions?” Journal of Animal and Plant Sciences 39, no. 2: 6411–6425.

[ece372984-bib-0042] Ruf, F. , S. Koné , and B. Bebo . 2019. “Le Boom de l'Anacarde en Côte D'ivoire: Transition Écologique et Sociale des Systèmes à Base de Coton et de Cacao.” Cahiers Agricultures 28: 21.

[ece372984-bib-0043] Sanon, Z. , J. T. Yameogo , H. Rabiou , and M. Hien . 2019. “Pression Anthropique et Dynamique des Peuplements de *Isoberlinia dok*a Craib et Stapf et *Isoberlinia tomentosa* (Harms) Craib et Stapf Dans le Domaine Soudanien du Burkina Faso.” International Journal of Biological and Chemical Sciences 13, no. 2: 911.

[ece372984-bib-0044] Schrauf, R. W. , and J. Sanchez . 2008. “Using Freelisting to Identify Access and Characterize Age Differences in Shared Cultural Domains.” Psychological Sciences and Social Sciences 63: 385–393. 10.1093/geronb/63.6.s385 19092048

[ece372984-bib-0045] Smith, S. E. , and D. J. Read . 2008. Mycorrhizal Symbiosis. Academic Press.

[ece372984-bib-0058] SODEXAM . 2024. “Données météorologiques des régions Bafing, Bagoué, Béré, Bounkani, Folon, Gontougo, Hambol, Iffou, Kabadougou, Poro, Tchologo et Worodougou.” https://sodexam.com.

[ece372984-bib-0046] Sokpon, N. , S. H. Biaou , C. Ouinsavi , and O. Hunhyet . 2006. “Bases Techniques Pour une Gestion Durable des Forêts Claires du Nord‐Bénin: Rotation, Diamètre Minimal D'exploitabilité et Régénération.” Bois et Forêts Des Tropiques 287, no. 1: 45–57.

[ece372984-bib-0047] Soro, G. , A. Yao , Y. Kouame , and T. Bi . 2017. “Climate Change and Its Impacts on Water Resources in the Bandama Basin, Côte D'ivoire.” Hydrology 4, no. 1: 18.

[ece372984-bib-0048] Tardío, J. , and M. Pardo‐de‐Santayana . 2008. “Cultural Importance Indices: A Comparative Analysis Based on the Useful Wild Plants of Southern.” 10.1007/s12231-007-9004-5.

[ece372984-bib-0049] Tiétiambou, F. R. S. , A. M. Lykke , G. Korbéogo , A. Thiombiano , and A. Ouédraogo . 2016. “Perceptions et Savoirs Locaux sur les Espèces Oléagineuses Locales dans le Kénédougou, Burkina Faso.” Bois et Forêts Des Tropiques 327, no. 1: 39–50.

[ece372984-bib-0050] Tonga Ketchatang, P. , L. Zapfack , L. P. R. Kabelong Banoho , and D. Endamana . 2017. “Disponibilité des Produits Forestiers Non Ligneux Fondamentaux à la Périphérie du Parc National de Lobeke.” VertigO 17: 173.

[ece372984-bib-0051] Traoré, L. 2013. Influence du Climat et de la Protection sur la Végétation Ligneuse de la Partie Occidentale du Burkina Faso, Thèse Unique, Université de Ouagadougou, 158 p.

[ece372984-bib-0052] Tsoumou, B. R. , K. J. Lumandé , J. P. Kampé , and J. D. Nzila . 2016. “Estimation de la Quantité́ de Carbone Séquestré par la Forêt Modèle de Dimonika (Sud‐ ouest de la République du Congo).” Revue Scientifique et Technique Foret et Environnement du Bassin du Congo 6: 39–45.

[ece372984-bib-0053] Van Cuyck, A. 2005. “Tests du Khi‐Deux, Corrélations, Variabilité. Pour une Méthodologie Quantitative, Réflexive et Structurale de L'étude des Champs, des Liens et des Relations.” Archive ouverte HAL Id: sic_00001500.

[ece372984-bib-0054] Vanié‐Léabo, L. P. L. 2016. “Ectomycorrhizal Fungi of Comoé National Park, a Biosphere Reserve in Northeast Côte d'Ivoire: Diversity, Fruiting Phenology and Production in Relation to Climate Variability.” PhD at University Félix Houphouet Boigny. 2016, 197 p.

[ece372984-bib-0055] Vroh, B. T. A. , A. Cissé , C. Y. Adou Yao , et al. 2015. “Relations Entre la Diversité et la Biomasse Aérienne des Espèces Arborescentes Dans les Agroforêts Traditionnelles à Base de Cacaoyers: cas de la Localité de Lakota (Côte D'ivoire).” African Crop Science Journal 23, no. 4: 311–326. 10.4314/acsj.v23i4.2.

[ece372984-bib-0056] White, F. 1986. “La Végétation de l'Afrique. Mémoire Accompagnant la Carte de la Végétation de l'Afrique Unesco/AETFAT/UNSO, OROTOM.”

[ece372984-bib-0057] Yogeeswari, N. , and S. Dharmarajan . 2005. “Betulinic Acid and Its Derivatives: A Review on Their Biological Properties, Perumal Current Medicinal Chemistry.” 12: 657–666. 10.2174/0929867053202214 15790304

